# GABA transaminase deficiency. Case report and literature review

**DOI:** 10.1002/ccr3.3753

**Published:** 2021-01-09

**Authors:** Amira Oshi, Abdullah Alfaifi, Mohammed Z. Seidahmed, Khalid Al Hussein, Abeer Miqdad, Abdelmohsin Samadi, Omar Abdelbasit

**Affiliations:** ^1^ Department of Pediatrics Security Forces Hospital Riyadh Saudi Arabia

**Keywords:** aminobutyrate aminotransferase, gamma aminobutyric acid, gamma aminobutyric acid transaminase, glutamic acid decarboxylase

## Abstract

GABA transaminase deficiency should be considered in the differential diagnosis of early onset epileptic encephalopathies. This case was diagnosed post‐mortem, but increased vigilance to this will allow for earlier diagnoses in other infants and families. This is a case study which involved diagnosis of a rare neurometabolic disorder in one of the babies in the family and eventual genetic counselling of the family. The family has been offered pre‐implantation genetic diagnosis for future pregnancies. This case reporting has been approved by the hospital research and ethical committee.

## Graphical abstract

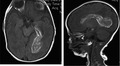

## 1 INTRODUCTION

Gamma‐aminobutyric (GABA) transaminase deficiency is a rare disorder with only few cases described in the literature. We present here a neonate who presented early with an epileptic encephalopathy. The recently described diagnostic criteria and gene sequencing are now the backbone for diagnosing this severe rare metabolic encephalopathy and have helped in understanding its metabolic effects and the pathophysiology. Affected families can benefit from genetic counseling for their future pregnancies. The variant in this baby (p.Gly106Ser) has not been described before.

In communities, where cousin marriage is common it is anticipated that inherited disorders will be seen more often. As expected the burden of recurrence of cases in one family can be great. This is true for cases with neurometabolic disorders where the accompanying physical and neurological handicap is high. The recent technological advances in genetic diagnosis has resulted in identification of rare neurometabolic disorders which enabled the medical profession to help these families. Our patient in this case report exemplifies the importance of utilization of whole exome sequencing in establishing the diagnosis and providing genetic counselling.

## 2 CASE REPORT

The proband, a term male newborn delivered after an emergency Caesarean section due to failure to progress.

The mother was para 6, gestational diabetic, and hypertensive. Antenatal ultrasound showed polyhydramnios but no apparent fetal anomalies. The parents are first cousins and had a female child with seizures and global developmental delay who died at the age of seven months.

The baby had good Apgar score and birth weight was 2650 grams (10th percentile for gestation). Head circumference was 34 cm while the length was 52 cm (both HC and length were on 25th percentile for gestation). The baby was sent to the normal newborn nursery.

At the age of six hours, the baby was noticed to be lethargic with poor feeding necessitating admission to neonatal intensive care unit (NICU). On clinical examination, the baby was hypotonic with dysmorphic features consisting of frontal bossing, hypertelorism, depressed nasal bridge, and deeply seated eyes. Primitive reflexes were absent but deep tendon reflexes were present. Other systemic examination revealed no abnormalities. The baby was treated for possible sepsis but went on to develop persistent seizures involving upper and lower limbs.

At this point, a diagnosis of an encephalopathy with seizures was entertained.

Investigations revealed a slightly elevated lactate of 3.42 mmol/L (N‐0.5‐2.2), normal ammonia, and unremarkable tandem mass spectrometry (TANDEM MS) for metabolic disorders. Other investigations including renal functions, liver functions, CK, coagulation profile, very long chain fatty acids, and chromosomal karyotype were normal.

EEG was done in awake/sleep state and showed a background rhythm consisting of fairly well developed activity of 4 Hz bilaterally synchronous and symmetrical. There were frequent spike and sharp waves seen independently multifocal mainly in bi‐temporal area. The record is consistent with multifocal epilepsy.

**FIGURE 1:**
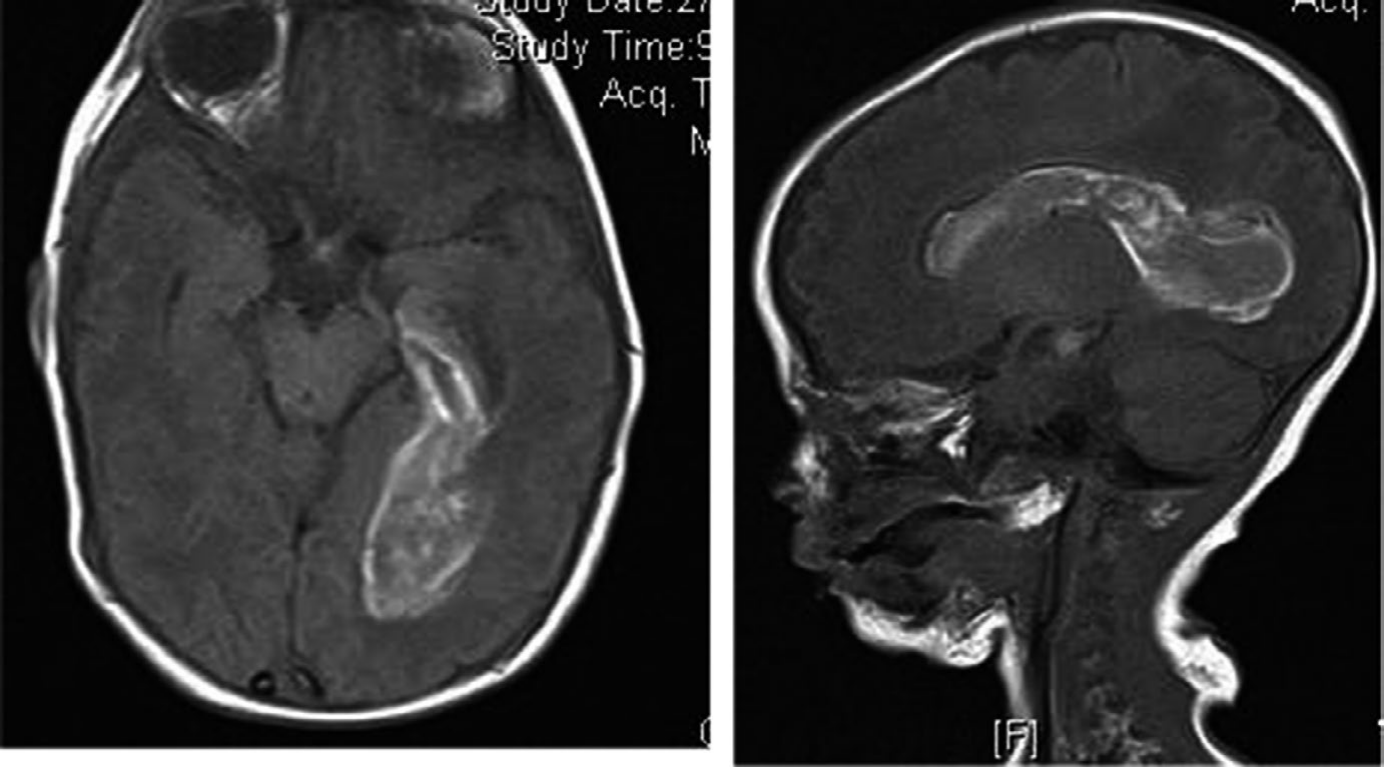
MRI of brain showing Intraventricular Hemorrhage

MRI at the age of 7 days showed extensive intraventricular hemorrhage. (Figure 1).

The baby was commenced on anticonvulsant therapy but continued to be lethargic, hypotonic with poor feeding, and swallowing incoordination.

At the age of 14 days, the baby had polyuria with hypernatremia of 159 mmol/L, hyperosmolality of 321 mOsm/kg (N‐270‐295), and urinary hypo‐osmolality, which prompted the diagnosis of diabetes insipidus. This was confirmed with a low‐serum antidiuretic hormone at less than 0.7 pg/mL (N‐1‐5) and which responded well to desmopressin.

With advancement of age the baby had developmental delay with microcephaly with occipito‐frontal diameter of 42.5 cm (<5th percentile for age), hypotonia with hyper‐reflexia, bilateral hearing impairment, cortical blindness and uncontrolled seizures, and eventually expired at the age of seven months.

Whole exome sequence confirmed presence of homozygous mutation in the *ABAT* gene with GABA transaminase deficiency. Both parents were heterozygous for the same mutation. (Table 1). The test for whole exome sequence was sent at the age of two weeks, but the result was not available until after the death of the baby.

**TABLE 1:** Whole exome sequence

Segregation analysis					
Variant no.	Gene	Chromosome	DNA change	Protein change	Zygosity
1	ABAT	Chr16_8844396G > A	c.316G > A	p.Gly106Ser	Homozygous
Confirmed by Sanger sequencing as a homozygous mutation in both directions in the index. Confirmed by Sanger sequencing as a heterozygous mutation in both directions in both parents.					

Sample	Sample ID	Genotype	Result	Flanking sequence	QC1	QC2
Index	1 166 452(S3363)	A/A	Homozygous	TGTCCCCATAGGTAAGAGCTG	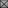	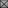
Father	557 776(S4276)	G/A	Heterozygous	TGTCCCCATAGGTAAGAGCTG	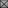	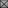
Mother	237 663(S4277)	G/A	Heterozygous	TGTCCCCATAGGTAAGAGCTG	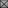	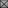

Note

Conclusion: Mutation identified in the ABAT gene.

Abbreviations: ABAT, Aminobutyrate transferase; Chr, chromosome; Gly, Glycine; Ser, Serine.

Consent was obtained from the family for the reporting of the case.

The case reporting was approved by the hospital research and ethical committee.

## 3 DISCUSSION

Our baby presented with early neonatal epileptic encephalopathy which proved on whole exome sequencing (WES) to have a novel homozygous missense/splice site donor variant in the 4‐aminobutyrate aminotransferase (*ABAT*) gene.

Gama aminobutyrate aminotransferase (GABA‐T) catalyzes the conversion of gamma‐aminobutyric acid (GABA) into succinic semialdehyde. GABA is the brain major inhibitory neurotransmitter with thirty to forty percent of cerebral synapses using it to facilitate inhibition.^1^ Only the excitatory neurotransmitter glutamate is more prevalent in the central nervous system. GABA results from the conversion of L‐glutamate via glutamic acid decarboxylase (GAD). GABA is then metabolized to succinic acid, which enters the tricarboxylic acid cycle where it is transaminated through alpha‐ketoglutarate. This forms a closed loop, which returns to glutamate and its conversion through GAD to GABA.^1^

GABA‐T deficiency is a rare disorder, which results in accumulation of GABA and beta‐alanine.^2^ The first cases were reported in 1984 by Jaeken et.al who described two consanguineous sibs presenting with severe hypotonia, psychomotor retardation, and hyperreflexia.^2^ The patient had accelerated linear growth associated with increased growth hormone. CSF showed high level of free GABA, homocarnosine, and beta alanine. The patient died at the age of 25 months while the brother who showed similar clinical features had died at the age of one year. Post mortem showed leukodystrophy.^2^

Medina‐ Kauwe et al described second unrelated patient with GABA aminotransferase deficiency. The phenotype in both included severe psychomotor retardation, hypotonia, hyperreflexia, lethargy, refractory seizures, and high pitched cry and EEG abnormalities. The second patient did not have accelerated linear growth. Brain MRI of the second patient showed agenesis of the corpus callosum, cerebellar hypoplasia, posterior fossa cyst and abnormal gyration. GABA concentration was significantly increased in all bodily fluids. Patient died at the age of five months.^3^ Since then Pearl et al. identified all previously reported cases of GABA transaminase deficiency and were able to describe the 10 cases of GABA‐T deficiency which have been reported in the literature.^4^ Nine cases had documented ABAT mutation. In this series all patients presented with neonatal/infantile onset encephalopathy, hypotonia and hypersomnolence. The median age at onset was 3 months (range 0‐7 months) with 4 having neonatal presentation.^4^

Our baby presented with the classical features described for GABA‐T deficiency including severe neonatal epileptic encephalopathy, lethargy, hypotonia, hyper‐reflexia, and poor feeding. Extensive intraventricular hemorrhage was seen on MRI (Figure 1). Whether this is a coincidental finding or related to the disorder is unclear.

Linear growth was on the 50th centile while weight and head circumference were below the 5th centile at the age of 7 months. Excessive linear growth was reported in other cases with elevated growth hormone^2^ which was not performed in our baby.

However, the other hormones including thyroid, adrenal and ACTH were normal.

Pituitary MRI was also normal. Central diabetes insipidus was diagnosed in our baby at the age of 14 days when he developed polyuria, hypernatremia, hyperosmolality associated with low antidiuretic hormone serum level. This responded well to desmopressin which was maintained until the death of the baby. Diabetes insipidus has not been reported before in GABA‐T deficiency but given the fact that the baby had severe epileptic encephalopathy, diabetes insipidus here is a unique factor and should be considered along with other systemic manifestations of this disorder outside the central nervous system.

Like the diabetes insipidus, the dysmorphism reported in our baby is not clear fit with the diagnosis. Whether this is an additional feature or suggest an additional process is to be noted here.

Like other reported cases of GABA‐T deficiency, autosomal recessive inheritance was confirmed in this family, both parents are heterozygous for the gene.

Recently homozygous mutations in *ABAT* have also been linked to a new form of mitochondrial DNA depletion syndrome which presents in combination with a neurometabolic disorder of GABA degradation that shares phenotypic overlap with individuals with mutations in *SUCLG1, SUCLA2,* and *ALDH5A1*.^5^ Mutations causing this type of ABAT deficiency lead to elevated levels of GABA in the brain as well as hallmarks of mitochondrial dysfunction in muscle.^5^ The clinical features in our baby may fit into this combination but mitochondrial testing would be required to assess for mitochondrial depletion syndrome.

In our search for a metabolic cause in our baby, we noticed that the plasma amino acids level for glutamate was high at 315 μmol/L (N‐ 20‐220), while the level for glutamine was low at 150 μmol/L (N‐230‐700). The major role of glutamine in the brain is that of a precursor of the neurotransmitter amino acids glutamate, aspartate and GABA. Disturbance of glutamine metabolism and/or transport contribute to changes in glutamic‐ergic or GABA‐ergic transmission associated with different pathological conditions of the brain which is best recognized in epilepsies.^6^ Kirby et al reported that amino acid analysis in patient tissues revealed significant elevation of aspartic acid and depletion of glutamine. The accumulation of GABA associated metabolites in patient tissues indicates significant disruption of fat, creatine and amino acid metabolism.^7^ That would explain why glutamine cannot be replenished leading to low serum glutamine level. On the other hand, since glutamate is the major excitatory neurotransmitter in the brain it is possible that the high level expressed in our baby may have played a role in the epileptic seizures seen.

The location of the ABAT gene is in chromosome 16. Known pathogenic variants reported in *ABAT* protein include missense substitution of leucine to phenylalanine, arginine to lysine, leucine to proline, arginine to glycine and asparagine to valine.^4^

Variants are classified based on specific criteria set out by the American College of Medical Genetics and Genomics (ACMG) into pathogenic, likely pathogenic, unclear significance, likely benign or benign. These criteria include reports and functional data about the specific variant, reports and functional data about other similar variants within specific gene, phenotype data, population data, and computational data.^8^ The detected variant in our case (p.gly106ser) causes a glycine to serine missense substitution within the *ABAT* protein. The homozygous G to A nucleotide alters the wildtype splice donor at the end of exon 5.^4^ This variant affects splicing and the resulting aberrant splicing lead to protein truncation and loss of function. The phenotype data also complied with the clinical features of GABA‐T deficiency. For these reasons we think that the variant in this baby (p.gly106ser) is pathogenic resulting in the phenotype of *ABAT* deficiency seen.

## CONFLICT OF INTEREST

None declared.

## AUTHORS CONTRIBUTION

Dr Omar Abdelbasit clinical management of the case and writing and revision of the manuscript. Dr Oshi A. active management of the case and literature review. Dr Alfaifi A. Genetisist who was involved in the management of the case, genetic counselling and review of the genetic part of the manuscript. Seidahmed MZ management and review of the neurological aspects of the case. Al Hussein K management of the case and revision of the manuscript. Miqdad A. Management of the case and the metabolic sequelae. Samadi A management and review of the endocrinological aspects.

## Data Availability

Data are available in article supplementary material.
